# Do More Active Children Sleep More? A Repeated Cross-Sectional Analysis Using Accelerometry

**DOI:** 10.1371/journal.pone.0093117

**Published:** 2014-04-02

**Authors:** Sheila M. Williams, Victoria L. Farmer, Barry J. Taylor, Rachael W. Taylor

**Affiliations:** 1 Department of Preventive and Social Medicine, University of Otago, Dunedin, New Zealand; 2 Department of Medicine, University of Otago, Dunedin, New Zealand; 3 Department of Women's and Children's Health, University of Otago, Dunedin, New Zealand; Universidad Europea de Madrid, Spain

## Abstract

**Aim:**

To determine whether levels of daytime physical activity are associated with sleep duration and night waking in children assessed using accelerometry, and if these associations change over time.

**Methods:**

24-hour accelerometry data were obtained from 234 children at 3, 5 and 7 years of age for at least 5 days at each time. Sleep duration was estimated using the Sadeh algorithm. Time spent in sedentary, light and moderate-vigorous (MVPA) activity was established using published cut-points. Appropriate statistical techniques were utilised to account for the closed nature of the data (24-hour periods).

**Results:**

Time spent asleep was related more to sedentary or light activity and not to MVPA. The most active (95^th^ percentile) children spent 55–84 fewer minutes asleep and 16–19 more minutes awake at night compared to the least active (5^th^ percentile) children. Children with later bedtimes slept less at night (30–40 minutes) and undertook more sedentary (10–15 minutes) but also more light (18–23 minutes) activity during the day. However, no differences in MVPA were apparent according to bedtime. Children slept slightly less on weekend nights (11 minutes) compared with week-nights, but only at 3 years of age. Most relationships were broadly similar at 3, 5 and 7 years of age.

**Conclusion:**

Children who are more physically active during the day have shorter total sleep time and are more awake at night than less active children. The protective effect of sleep on obesity does not appear to be mediated by increased physical activity.

## Introduction

The observation that inadequate sleep is a strong risk factor for child obesity [1], [2] has underscored the importance of determining what might be explaining this effect. One hypothesis is that higher amounts of physical activity promote better sleep, which makes intuitive sense. Several studies investigating whether physical activity influences sleep in children and adolescents and vice versa have used questionnaires or time-use diaries to assess either or both behaviours. Inconsistency in findings is apparent with studies reporting that physical activity promotes better sleep [3], has no relationship with sleep duration [4], [5], or alternatively, is associated with less sleep [6]. However, neither sleep nor physical activity are assessed particularly well by questionnaire, an issue that is further complicated in young children for whom assessment by parental proxy is necessary [7], [8], [9]. The advent of actigraphy has provided a relatively simple technique for estimating both sleep and physical activity by objective means [10], [11]. However, despite growing interest in the relationships between sleep, physical activity and energy metabolism [12], few datasets exist which have used actigraphy over 24-hour periods to objectively measure both behaviours. This is important given that relationships between physical activity and sleep with health differ according to whether behaviour is measured using objective or subjective tools [13].

Two large studies with accelerometer measures of physical activity and questionnaire estimates of sleep reported no significant relationship between minutes of moderate-vigorous physical activity (MVPA) and sleep duration [14], [15]. Only two studies appear to have examined both physical activity and sleep using accelerometers. One reported no relationship between total sleep time and physical activity measured over a one-week period [16]. The second study used temporal analyses and showed that being active during the day decreased rather than increased sleep duration that night. Similarly, a longer sleep was associated with *less* physical activity the following day [17].

There is also interest in measures of sleep quality rather than sleep duration per se. It could be that the time the child goes to bed rather than the duration itself is a more important determinant of physical activity [18]. Although MVPA has been associated with shorter sleep latency (time taken to go to sleep) [19], the actual difference in latency was only 1 minute per every 100 increase in counts per minute, a difference that is unlikely to be clinically important. Furthermore, measurements were only obtained on one day and sleep latency is a particularly unstable measure, requiring multiple days to ensure reliability (Taylor, unpublished data). Others have reported that greater participation in MVPA promotes better sleep efficiency (less night awakening) within an individual child. However when analysed on a group basis, increasing MVPA seems to be related to *more* fragmented sleep [16].

The observation that sleep is protective against obesity whereas time spent sedentary is a risk factor for obesity, means that determining the relationship between sleep and sedentary time is also of interest. Two studies have indicated that children or adolescents reporting greater sleep time by questionnaire display less time in sedentary behaviour measured using accelerometry [14], [15]. However others have also shown that total sleep time is not associated with sedentary time when both behaviours are measured using accelerometry [16].

Given the uncertainties and limitations of the existing data and the lack of any published longitudinal analyses, determining how sleep is related to all types of physical activity (light, moderate, vigorous) and inactivity (sedentary time) is of considerable interest. However, this analysis is complicated by the closed nature of the 24-hour data. If one variable (e.g. sleep) increases, then another variable within the same 24-hour block *must* decrease (e.g. sedentary, light or MVPA). Thus standard multivariate techniques cannot be used for compositional data of this kind. The aims of our study were 1) to determine the relationships between sleep duration and time awake at night, and time spent in sedentary, light and MVPA in children and 2) to ascertain if these relationships differed at 3, 5 and 7 years of age.

## Methods

The FLAME (Family Lifestyle, Activity, Movement and Eating) project was a longitudinal observational study designed to examine factors in early childhood which may contribute to the development of overweight and obesity. Children were recruited just before their third birthday in 2004/5 from a cohort born at the only maternity hospital servicing the city of Dunedin, New Zealand (Queen Mary Maternity). Exclusion criteria included prematurity (<37 completed weeks gestation), multiple births, major congenital abnormalities, severe postnatal illness or living in families unlikely to be resident locally for the next two years. After exclusions, 413 children were eligible, of which 244 (44% girls) agreed to participate (59% response rate). The children were predominantly European (83%), with 10.8% identifying as Maori, 3.7% as Pacific Islanders and 2.5% as other ethnicities, reflecting the local population (Statistics New Zealand, 2001). The study received ethical approval from the Lower South Regional Ethics Committee (Reference OTA/04/03/023) and signed informed consent was obtained from the parents or guardians of each participating child.

### Data collection

Children were seen six-monthly at University research clinics between ages 3 and 7 as close as possible to their birthdays, although not all measures were obtained at all time-points. Although accelerometry data were obtained at 6 time-points within the 4 years of follow-up, only data from 3, 5 and 7 years of age are presented in this paper for simplicity.

Height was measured in duplicate to the nearest 0.1 cm using an electronic wall-mounted stadiometer (Heightronic, QuickMedical, WA, USA) and weight by electronic scales (Mettler Toledo, USA) in duplicate to the nearest 0.1 kg. Body mass index (BMI) was calculated (kg/m^2^) and overweight and obesity were defined according to CDC reference cutoffs [20]. Physical activity and sleep were measured using omnidirectional Actical accelerometers (Actical, Mini-Mitter, Bend, OR) attached by belts to the waist. Parents were instructed to keep the monitors on the children at all times for at least five consecutive days, including two weekend days. Epoch length was set at 15 seconds to account for the intermittent and sporadic nature of young children's activity [21]. Data were excluded if the total counts for the day were less than 10,000 or more than 20 million, which may indicate accelerometer malfunction [22]. Parents completed an activity log describing when the children went to bed and woke up each day of measurement. The Sadeh sleep algorithm within the ActiLife programme (ActiLife 6, Actigraph, Pensacola, FL) was used to estimate sleep duration for each night for each child. The time the child went to bed and woke up is then used as a starting point to estimate the actual time the child went to sleep (sleep onset) and woke up (sleep offset), from which total sleep duration is calculated. As these bed and wake times differ from day to day both within and between children, this individual level data were used to “remove” sleep from the 24-hour accelerometry data using MeterPlus (MeterPlus, Santech, Inc., San Diego, CA), a commercial data reduction programme. This means that our measures of physical activity are obtained from awake time only, individual to each day in each child. An additional filter of non-wear time (at least 20 consecutive minutes of zero counts [11]) was also included. Time spent in sedentary (0–47 counts per minute), light (48–2031 counts per minute), moderate (2032–2875 counts per minute) and vigorous (≥ 2876 counts per minute) physical activity (PA) was calculated using the cut-points of Evenson et al [23] developed in children aged 5 to 8 years.

### Statistics

A day and a night, taken from the time the child awoke to the time the child awoke the following day was counted as valid if data were recorded for more than 20 hours. In practice, data was recorded for between 20 and 29 hours. The data were recorded as counts, before being divided into the amount of time spent at each intensity of activity. As accelerometers are worn for a 24-hour day, each day represents a closed dataset i.e. individual components (sedentary, light, moderate, vigorous, sleep and awake at night) are *not* independent because if more minutes can be attributed to one intensity of activity, fewer minutes *must* be attributed to one or more of the other components. In this analysis we have obtained the proportion of time allotted to each activity or component by dividing the time for each component by the total recording time. Thus the sum of the six components used in our analysis is always 1. Data of this kind have a number of in built problems as the components are not independent. This means that at least one element of the correlation matrix of these components must be negative but it is not clear whether this represents a genuine negative association or whether it is a statistical artefact. Thus data of this type cannot be analysed using standard multivariate methods designed for unconstrained multivariate data. The data are, therefore, a quantitative description of the composition of activity over some time period and convey relative information which is best thought of in terms of ratios or log ratios. If the ratio or log ratio of two components is consistent for all sets of observations, its standard deviation or variance will be small and the relationship will be consistent across the population. Conversely if the ratio of the components is very different for different sets of observations its variance will be large and the relationship inconsistent.

In order to visualise the data, as part of an exploratory analysis we constructed biplots, after transforming the data using the centred transformation [24]. Thus the six part composition (sedentary, light, moderate, vigorous, sleep and awake at night) can be transformed into 6 ratios between the components and their geometric average represented by x_j_/g(x) for j = 1,2,3,4,5,6. The geometric mean is g(x) = (x_1_.x_2_.x_3_.x_4_.x_5_.x_6_)^1/6^. The log of the transformed variables, the sum of which is zero, is used for the biplots. A biplot is a way of presenting data on both the participants and the variables at the same time. The samples are displayed as points and the variables as rays or vectors. In the interests of clarity only the plot for the variables is presented. Distances between the ends of the rays of two variables approximate the standard deviations of their log-ratio. Thus rays where the ends are close together provide an indication of a consistent multiplicative relationship between the two variables. Conversely, those with rays in different directions imply little relationship between the two variables. As this analysis is akin to a principal components analysis, it is possible to form two independent linear combinations or dimensions of the variables. The weights for these dimensions are represented by the projections of the rays on the horizontal and vertical axes. The proportion of the variance of the original variables explained by these dimensions provides an indication of how well the dimensions represent the data and are expressed as % explained on the biplots presented in this paper.

A number of approaches to analyse data of this type in a more formal way have been proposed. One of the most flexible is the ‘additive’ transformation in which all but one of the components are expressed as a ratio of it and the last component and then logged. In this case as moderate and vigorous activity were combined, there were 5 (rather than the original 6) components. These were transformed into 4 log ratios:
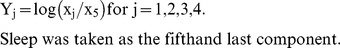



This transformation has been incorporated in the Stata estimation procedure fmlogit (Buis) for a fractional multinomial logit model [25], a generalisation of a fractional logit model proposed by Papke and Wooldridge [26]. The model, which is fitted using quasi-maximum likelihood, included the five components, sleep being entered last, as dependent variables and restrains the sum of the predicted proportions to 1. As the children had more than one set of observations at each age, the sandwich estimator was used to adjust the standard errors. Pearson residuals were used to examine the fit of the model. The data were analysed separately for each age.

It is possible to back transform the parameters from the model to obtain estimates of the proportions of each component (e.g. sleep) at various values of the independent variable of interest (e.g. overall activity). We were particularly interested in the association with counts per minute. As its distribution was very skewed, counts per minute was log transformed and we then estimated the proportions of sleep, MVPA, light and sedentary activity and time awake at night at the 5^th^ and 95^th^ centile of counts per minute. We have also estimated the change in the proportions commensurate with the difference between the 5^th^ and 95^th^ percentiles of counts per minute. This difference was converted to hours assuming a 24-hour time period. Data were analysed using Stata (Stata 12.0, StataCorp, College Station, TX).

## Results

This study included 234 children (134 boys, 100 girls). Accelerometry data were available for 120 boys and 96 girls at age 3, 119 boys and 86 girls at age 5 and 100 boys and 67 girls at age 7. The means and standard deviation for the variables of interest are shown in Table 1. 40.7% of children were overweight or obese at 3 years of age, which reduced to 21.1% at 7 years. Non-wear time was very low among our children, with data available for an average of 23.6 (1.0) hours each day. Children spent 60 (at age 3) to 90 (age 7) minutes a day in MVPA, plus several more hours in activities at light intensity.

**Table 1:** Time spent in sleep and physical activity at 3, 5 and 7 years of age.

		Age (years)		
		3	5	7
n		216	205	167
Male n (%)		120 (56%)	119 (58%)	100 (60%)
Height (cm)		95.5 (3.72)	109.8 (2.40)	121.8 (5.27)
Weight (kg)		15.6 (1.79)	19.8 (2.40)	24.8 (3.64)
BMI (kg/m^2^)		17.1 (1.25)	16.4 (1.31)	16.6 (1.64)
Accelerometer data	Total counts (24 hours)	681,556 (211,605)	413,612 (182,414)	416,226 (196,669)
	Time worn (hours/day)	23.4 (0.5)	23.7 (0.4)	23.6 (0.4)
	Counts per minute^1^	484 (150)	291 (128)	293 (137)
Sleep	Sleep (hours/day)	10.2 (0.7)	10.7 (0.6)	10.6 (0.6)
	Time awake at night (hours/day)	0.8 (0.5)	0.5 (0.3)	0.4 (0.3)
Physical activity	Sedentary activity (hours/day)	3.3 (0.9)	5.7 (0.9)	6.8 (1.4)
	Light activity (hours/day)	7.5 (0.9)	6.0 (0.8)	4.8 (1.3)
	MVPA (hours/day)	1.6 (0.7)	0.9 (0.6)	1.0 (0.5)
Log transformed ratios	MVPA to sleep	−2.01 (0.48)	−2.71 (0.60)	−2.57 (0.51)
	Light activity to sleep	−0.32 (0.14)	−0.59 (0.17)	−0.83 (0.30)
	Sedentary activity to sleep	−1.18 (0.32)	−0.66 (0.26)	−0.47 (0.24)
	Time awake at night to sleep	−3.03 (0.80)	−3.83 (0.80)	−4.00 (0.90)

Data are presented as mean (SD).

MVPA is moderate-to-vigorous physical activity.

1 Counts per minute refer to 24-hour data not awake time only.

Because the nature of the data meant that simple correlations were difficult to interpret, we examined *relative* associations among pairs of components. The standard deviations of the log-transformed ratios of physical activity at different intensities (sedentary, light, MVPA) to sleep are of particular interest (Table 1). The smallest standard deviations are for the ratios of light (or sedentary) activity to sleep, which range from 0.14–0.32, showing that the most consistent multiplicative associations were between these variables. In actual terms, the proportion of time 3-yr old children spent in light activity was about 73% of the time they spent asleep. In contrast, the standard deviations for the ratios of awake at night to sleep were much larger indicating that the relationship between being awake at night and total sleep was much less consistent. The average proportion of time children spent awake at night was 5% of the time they spent asleep. Similarly, the relationship between MVPA and sleep was inconsistent across different levels of overall activity (expressed as counts per minute).

**Figure 1:**
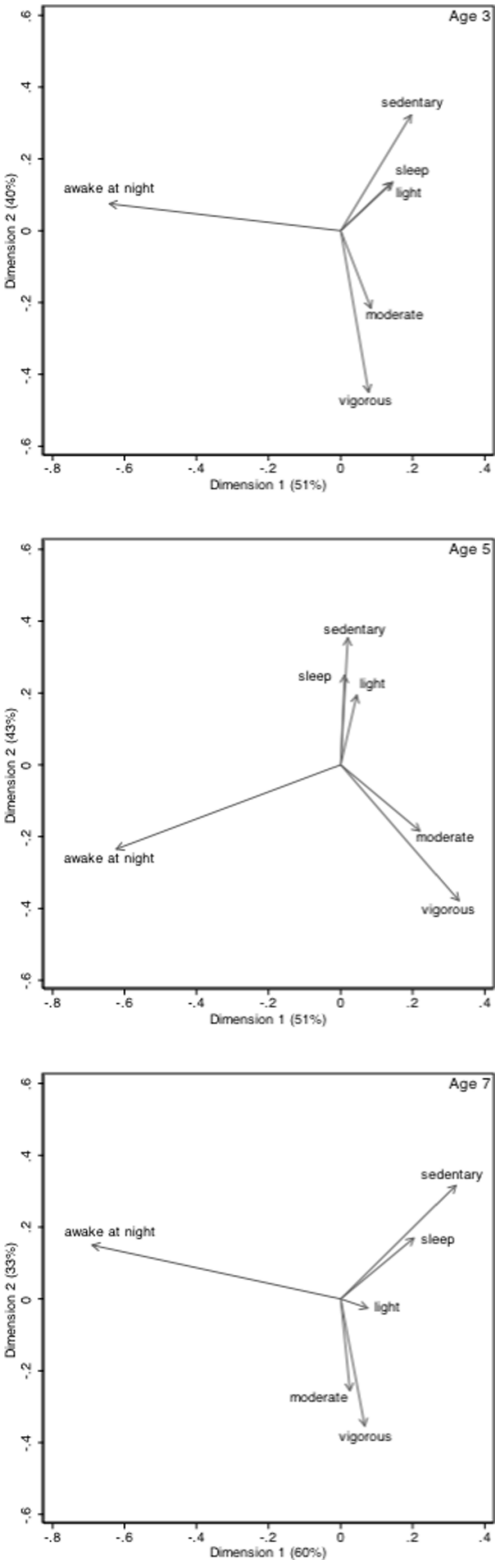
Biplots of the relationships between time spent asleep at night, awake at night, and in sedentary, light, moderate, and vigorous activity during the day at 3, 5 and 7 years of age.

The relationships among the six components are also illustrated in the biplots in Figure 1. These further demonstrate that time asleep at night appears to be related to time spent in sedentary and/or light activities, whereas there is little relationship with time in MVPA during the day. Time awake at night does not appear to be related to overall sleep duration or activity at any intensity during the day. The biplot for age 3 explains 91% of the variance in the original data, with the first and second dimensions (linear combinations of the original variables) explaining 51% and 40% of the original variance respectively. Similar values were observed at 5 and 7 years of age.

**Figure 2:**
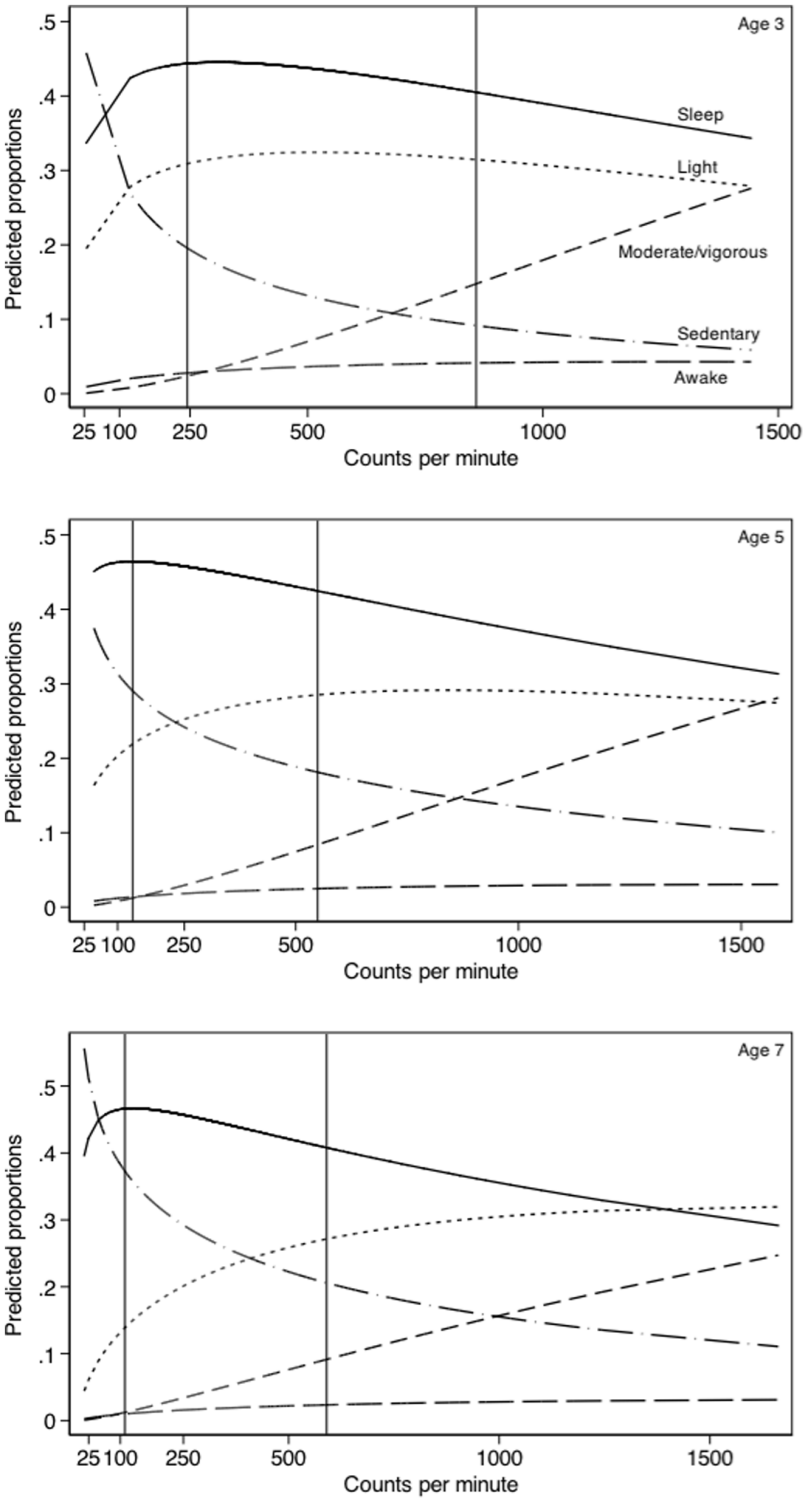
Proportion of time spent in sedentary, light, and moderate-vigorous physical activity, and sleep and time awake at night in relation to overall physical activity (counts per minute).

Because the relation between moderate and vigorous activity was consistent at all three ages and their contribution to the composition was small compared with those for sleep and sedentary or light activity, they were combined for further analysis. The association between the proportion of each activity and overall physical activity (as counts per minute) are illustrated in Figure 2. Given the uncertainty surrounding the activity level at the extremes of the distribution, most attention should be focused on the 90% of predicted values lying between the 5^th^ and 95^th^ centiles, indicated by the two vertical lines on each graph. Figure 2 demonstrates that at age 3, the ratio of light activity to sleep is fairly constant, indicating that the association does not depend on overall activity (counts per minute). As children become more active, sleep and sedentary activity tends to decline and MVPA increases. Patterns at 5 and 7 years of age are broadly comparable.

These differences in the amount of time spent asleep or awake at night and in different intensities of activity are quantified for the least (5^th^) and most (95^th^) active children in Table 2. This table demonstrates that the most active children spent 0.92 hours (55 minutes) less time asleep at night compared with the least active children at 3 years of age. Similar figures of 1.02 hours and 1.40 hours were observed at 5 and 7 years respectively. Interestingly, more active children were also awake more at night, for 16–19 minutes. These children spent less time in sedentary activity (10% or 2.49 hours at age 3) and more time in light (1% or 0.14 hours) and MVPA (12% or 2.95 hours). Similar patterns were observed at 5 and 7 years of age.

**Table 2:** Change in time spent in each category of activity (sleep, awake at night, sedentary, light, MVPA) in the least to most active children (change in counts per minute from the 5^th^ to the 95^th^ percentile).

		Proportion of the day in each category of activity			
Age	Variable	Estimate (SE) at the 5^th^	Estimate (SE) at the 95^th^	Difference between the estimates as a proportion (95% CI)	Difference between the estimates in hours (95% CI)
3	Sleep	0.44 (0.003)	0.41 (0.004)	−0.04 (−0.05, −0.03)	−0.92 (−1.16, −0.68)
	Awake at night	0.03 (0.002)	0.04 (0.003)	0.01 (0.01, 0.01)	0.32 (0.17, 0.47)
	Sedentary time	0.20 (0.004)	0.09 (0.002)	−0.10 (−0.11, −0.10)	−2.49 (−2.66, −2.31)
	Light activity	0.31 (0.004)	0.31 (0.004)	0.01 (−0.01, 0.02)	0.14 (−0.14, 0.42)
	MVPA	0.02 (0.0004)	0.15 (0.002)	0.12 (0.12, 0.13)	2.95 (2.88, 3.01)
5	Sleep	0.46 (0.004)	0.42 (0.005)	−0.04 (−0.05, −0.03)	−1.02 (−1.26, −0.79)
	Awake at night	0.01 (0.001)	0.03 (0.002)	0.01 (0.01, 002)	0.27 (0.17, 0.37)
	Sedentary time	0.29 (0.004)	0.18 (0.003)	−0.11 (−0.12, −0.10)	−2.69 (−2.87, −2.52)
	Light activity	0.22 (0.003)	0.23 (0.005)	0.07 (0.06, 0.08)	1.61 (1.41, 1.81)
	MVPA	0.01 (0.002)	0.09 (0.001)	0.08 (0.07, 0.08)	1.83 (1.79, 1.88)
7	Sleep	0.47 (0.003)	0.42 (0.004)	−0.06 (−0.07, −0.05)	−1.40 (−1.64, −1.16)
	Awake at night	0.01 (0.004)	0.02 (0.002)	0.01 (0.01, 0.02)	0.32 (0.22, 0.43)
	Sedentary time	0.37 (0.003)	0.21 (0.006)	−0.17 (−0.18, −0.16)	−4.01 (−4.28, −3.73)
	Light activity	0.14 (0.002)	0.27 (0.006)	0.13 (0.12, 0.14)	3.18 (2.96, 3.40)
	MVPA	0.01 (0.0001)	0.09 (0.001)	0.08 (0.08, 0.08)	1.90 (1.86, 1.93)

MVPA is moderate-to-vigorous physical activity.

The associations between time in different activities and demographic variables, time the child went to bed at night, and weekend/weekday variation are shown in Table 3. The activity patterns were strongly associated with bedtime at all three ages. Children who went to bed later slept 30–40 minutes less each night and were more sedentary (10–15 minutes per day). However these children also participated in 18–23 more minutes of light activity each day whereas no differences were observed in MVPA. Children slept less (11 minutes) on weekend days compared with weekdays at age 3, but no differences were observed at older ages. In general, weekends were associated with less sedentary activity (5 and 7 years) and more light activity (3 and 7 years).

**Table 3:** Correlates of the difference in time (minutes) for each variable of interest (time asleep and awake at night, and intensities of physical activity).

		Sleep variables		Physical activity categories			
Age		Sleep (minutes)	Awake at night (minutes)	Sedentary (minutes)	Light (minutes)	MVPA (minutes)	Overall P†
3	Sex (male)	−9.2 (5.3)	9.9 (4.0)	−3.3 (7.8)	−1.4 (6.8)	4.0 (6.2)	0.150
	Maternal education (tertiary)	3.0 (5.8)	0.4 (4.6)	14.7 (7.9)	−12.2 (7.1)	−5.9 (6.1)	0.290
	First born (yes/no)	4.6 (6.6)	1.1 (5.5)	8.1 (8.6)	−13.4 (7.6)	−0.4 (7.2)	0.520
	Smoking in pregnancy (yes/no)	−6.1 (7.1)	9.2 (6.1)	−18.0 (9.5)	−1.4 (8.9)	16.4 (9.4)	0.142
	Bedtime (hours)^1^	**−30.4 (2.7)**	**−7.3 (2.3)**	**15.0 (3.0)**	**17.6 (3.2)**	5.2 (2.9)	<0.001
	Weekend (vs weekday)	**−11.2 (3.6)**	2.2 (1.3)	−4.8 (3.7)	**14.1 (3.7)**	−0.4 (3.2)	<0.001
5	Sex (male)	**−10.7 (5.3)**	1.7 (2.5)	−9.2 (8.1)	**15.6 (6.5)**	2.5 (2.8)	0.138
	Maternal education	5.8 (5.2)	**−5.6 (2.7)**	**30.4 (8.1)**	**−20.3 (6.3)**	−10.2 (5.3)	0.002
	Bedtime (hours)^1^	**−35.6 (2.2)**	**−4.0 (1.3)**	**10.1 (3.0)**	**21.6 (2.9)**	7.9 (1.3)	<0.001
	Weekend (vs weekday)	−1.0 (3.0)	2.2 (1.6)	**−9.7 (4.0)**	6.2 (2.9)	2.3 (1.7)	0.112
7	Sex (male)	**−16.4 (6.1)**	0.4 (2.7)	8.5 (13.8)	−1.6 (12.2)	9.1 (5.0)	0.002
	Maternal education	1.4 (6.6)	0.1 (2.9)	21.3 (14.1)	−15.1 (12.4)	−7.5 (5.2)	0.401
	Bedtime (hours)^1^	**−39.0 (2.2)**	**−4.0 (1.3)**	**14.7 (4.2)**	**23.2 (3.6)**	**5.3 (1.4)**	<0.001
	Weekend (vs weekday)	0.9 (3.3)	−1.6 (1.4)	**−16.4 (4.9)**	**16.1 (4.3)**	1.0 (2.2)	0.002

Data are presented as estimate (standard error) and significant effects are shown in bold. MVPA is moderate-to-vigorous physical activity.

1 Change in predicted dependent variables when bedtime moves from half an hour below the mean to half an hour above the mean.

† Test of significance for the whole model.

## Discussion

Using appropriate statistical techniques, our study demonstrates that children with higher levels of overall activity, measured as counts per minute, have lower total sleep time and greater time awake at night than less active children. Further analysis by intensity of activity demonstrated that sleep duration was not related to time in MVPA. Although sleep duration appeared to be related to increases in time in light activity, this was not enough to offset the corresponding increases in sedentary time, resulting in the negative relationship between sleep duration and physical activity overall.

Although there is widespread belief that encouraging a child to be physically active will make them sleep better at night, our findings support most existing research in children, which has found no observable relationship [4], [5], [14], [15], [16] or that the opposite is true [6], [17]. Several theories are possible for these findings. Firstly, it may simply be that there are only a certain number of hours in the day, and sleeping longer produces an overall time deficit meaning that time in other activities must reduce [18]. Alternatively, it is conceivable that the lack of an effect is due to sleep and physical activity being typically measured over multiple days with average values used in analyses. Thus individual days with high activity are not generally compared with sleep that night (or vice versa) and any potential relationships are obscured within average values. This is supported by the findings of Ekstedt et al [16] who showed that greater participation in MVPA promotes better sleep efficiency (less night awakening) *within* an individual child. However when analysed on a group basis, increasing MVPA was related to *more* fragmented sleep [16]. The only study that appears to have directly examined temporal relationships between activity and sleep also showed that being active during the day decreased rather than increased sleep duration that night. The converse was also true; a longer sleep was associated with *less* physical activity the following day [17]. However, temporal analyses such as this are still limited by the closed nature of the data, as 24-hour periods are still effectively being examined. A final explanation could be statistical in that relationships between sleep, light and sedentary only occur because they represent the biggest proportions of time during the day and therefore significant relationships are more likely to be observed. However, this is unlikely as we examined the logs of the ratios which should negate that explanation.

It may be that physical activity promotes *better* sleep rather than *more* sleep, that is, sleep quality rather than quantity is improved. Measures of sleep quality include sleep latency (length of time it takes to go to sleep after going to bed), and the amount of time spent awake during the night (after sleep has commenced). However, to date, few studies have examined sleep quality in relation to physical activity [6], [16], [19]. In our sample, having a later bedtime consistently reduced total sleep time in our children by 30–40 minutes a night and only a small proportion of this deficit was regained through significantly shorter wake after sleep onset in these children (4–7 minutes a night). This seems feasible given that variation in wake up time is less possible due to school and work commitments, even perhaps in such young children. Others [6] have also shown that later bedtime reduces sleep duration overall in older children. Late-risers also had greater odds of insufficient levels of MVPA (<60 minutes a day) [6]. Our findings are quite different in that we observed no effect of a later bed-time on MVPA, but an *increase* in light activity as well as more sedentary time. These differences may have arisen due to variation in assessment (time-use diaries and pedometers versus accelerometry) or analysis between the two studies.

The strengths of our study include the use of multiple-day accelerometry to assess both sleep duration and physical activity in a reasonable sized sample of children, the inclusion of several time periods to assess whether patterns differed as the children aged, and the use of appropriate statistical techniques to manage this complex data. However, there are also limitations inherent in accelerometry analysis. Accelerometry does not measure sleep per se, but rather estimates sleep from the absence of physical activity. Although comparisons with polysomnography, the gold standard for assessing sleep, have generally supported the use of actigraphy for the determination of sleep onset, the assessment of wake time and awakenings during the night is more problematic [27], [28]. While our overall retention from 3 to 7 years of age was reasonable at 77%, there is potential bias in the later data from missing cases. This seems unlikely however as children who later dropped out of the study did not differ from those remaining in the study for age, sex distribution or initial sleep or activity patterns at 3 years of age (data not shown). Classification of activity into different intensities of activity is also dependent on which threshold values are chosen. Unfortunately appropriate cut-point values for the Actical accelerometer do not exist for the entire age range under study. Thus we chose cut-points that had been validated in children aged 5 to 8 years only [23].

In conclusion, more active children do not sleep more than less active children and the reverse may even be true. Ensuring children go to bed at night at an appropriate time offers a simple tool for promoting adequate sleep, and may reduce sedentary time during the day, but does not appear to impact on time in more vigorous activities.
